# *Caesalpinia Sappan* Wood Extract Alleviates Ferroptosis‐Like Oxidative Injury and Restores Macrophage Polarization Balance in Iron Overload–Induced Liver Fibrosis in Rats

**DOI:** 10.1155/cjgh/3174951

**Published:** 2026-09-03

**Authors:** Mohammad Ghozali, Jeri Nobia Purnama, Desak Made Malini, Erick Khristian, Ratu Safitri

**Affiliations:** ^1^ Department of Biomedical Sciences, Faculty of Medicine, Padjadjaran University, Jatinangor West Java, 45363, Indonesia, unpad.ac.id; ^2^ Study Program of Biotechnology, Post Graduate School, Padjadjaran University, Dipati Ukur No. 35, Bandung, Indonesia, unpad.ac.id; ^3^ Department of Biology, Faculty of Mathematics and Natural Sciences, Padjadjaran University, Jatinangor West Java, 45363, Indonesia, unpad.ac.id; ^4^ Study Program of Medical Laboratory Technology, Faculty of Health and Science Technology, Jenderal Achmad Yani University, Cimahi West Java, 40531, Indonesia

**Keywords:** brazilin, *Caesalpinia sappan*, ferroptosis, iron overload, liver fibrosis, macrophage polarization

## Abstract

Iron overload–induced liver fibrosis involves interconnected pathological drivers: ferroptosis‐like oxidative injury and dysregulated macrophage polarization. Although *Caesalpinia sappan* L. possesses well‐characterized antioxidant and anti‐inflammatory properties, its efficacy against this dual pathological axis has not been experimentally established. This study investigated the hepatoprotective effects of *C. sappan* wood extract (SWE) in an iron dextran–induced rat model of liver fibrosis. Male Wistar rats received intraperitoneal iron dextran (cumulative dose of 180 mg/kg BW over 39 days) to induce hepatic iron overload, followed by 28‐day oral administration of SWE (25, 50, 75, or 100 mg/kg BW/day) or deferasirox (20 mg/kg BW/day). Hepatic iron concentration, liver function markers, oxidative stress parameters (MDA, SOD, and GPx4), macrophage polarization markers (CD86 and CD163), inflammatory and fibrogenic mediators (IL‐6, IL‐10, TGF‐β1, and MMP‐12), and fibrosis severity were assessed through biochemical, immunohistochemical, and histopathological analyses. SWE was standardized to 25.12% w/w brazilin by HPLC–PDA. SWE showed dose‐related effects with optimal efficacy at intermediate doses, reducing hepatic iron burden, restoring GPx4 activity, suppressing lipid peroxidation, and enhancing antioxidant defense, collectively attenuating ferroptosis‐like oxidative injury. Concurrently, SWE reduced M1 macrophage activation (decreased CD86 and IL‐6) while promoting M2 polarization (increased CD163 and IL‐10), suppressed TGF‐β1‐driven fibrogenesis, and restored MMP‐12–mediated matrix remodeling, resulting in significant reductions in collagen deposition and fibrotic area. SWE performed comparably to deferasirox across most endpoints, with superior MMP‐12 recovery. SWE simultaneously attenuates ferroptosis‐like oxidative injury and restores macrophage polarization balance, establishing it as a mechanistically coherent multitarget candidate for iron overload–induced liver fibrosis with translational potential beyond that of iron chelation alone.

## 1. Introduction

Liver fibrosis affects hundreds of millions of people worldwide and progressively advances through successive stages toward cirrhosis, portal hypertension, and hepatocellular carcinoma, constituting a leading cause of liver‐related mortality globally [1, 2]. Despite advances in the clinical understanding of fibrogenesis, no pharmacological agent has yet demonstrated reliable efficacy in halting or reversing established fibrosis, underscoring the continued clinical need for therapies that target its underlying cellular and molecular mechanisms [3].

Among the various causes of chronic hepatic injury, iron overload represents a mechanistically distinct and clinically significant contributor to liver fibrosis, arising from hereditary hemochromatosis or long‐term transfusion dependence in disorders such as *β*‐thalassemia, in which up to 65%–75% of patients develop hepatic fibrosis correlating with transfusional iron burden [4–7]. At the molecular level, excess iron catalyzes reactive oxygen species generation through Fenton‐type chemistry, driving lipid peroxidation and hepatocellular injury that predispose hepatocytes to ferroptosis, an iron‐dependent form of cell death defined by suppressed glutathione peroxidase 4 (GPx4) activity [8, 9]. This ferroptotic injury triggers Kupffer cell activation and macrophage recruitment, shifting polarization toward proinflammatory M1 phenotypes (IL‐6) and away from reparative M2 phenotypes (IL‐10, CD163, and MMP‐12), thereby amplifying TGF‐β signaling and hepatic stellate cell–driven fibrogenesis [10–15]. This dual ferroptosis–macrophage polarization axis, therefore, provides a mechanistically coherent and experimentally tractable model for evaluating therapeutic interventions in iron‐driven liver fibrosis, with restoration of polarization balance toward an M2‐dominant state representing a rational therapeutic objective [8].

*Caesalpinia sappan* L., locally known as kayu secang and widely used in Southeast Asian traditional medicine [16], possesses well‐characterized antioxidant and anti‐inflammatory properties attributable principally to its major bioactive constituent, brazilin, which has been reported to scavenge reactive oxygen species [17, 18] and modulate cytokine production and immune cell polarization [19]. However, despite this established pharmacological profile, no study has experimentally tested whether *C. sappan* wood extract (SWE) can simultaneously target both the ferroptotic oxidative injury and the macrophage polarization dysregulation that jointly drive iron overload–induced liver fibrosis.

Building on our previously validated iron overload rat model and mechanistic framework [8], the present study investigated whether SWE can simultaneously attenuate GPx4‐mediated ferroptotic injury and redirect macrophage polarization from M1 toward M2 phenotypes, thereby reducing hepatic dysfunction and fibrotic progression. Using an integrated biochemical, histopathological, immunohistochemical, and cytokine‐based approach, this study aims to establish the mechanistic basis of *C. sappan*–mediated hepatoprotection and to evaluate its translational potential as a multitarget therapeutic candidate for iron overload–associated liver fibrosis, addressing a gap in natural product–based interventions for this dual pathological axis.

## 2. Materials and Methods

### 2.1. Chemicals and Reagents

Iron dextran (ID) was purchased from Sigma‐Aldrich (St. Louis, MO, USA). Deferasirox (Kalbe Farma, Jakarta, Indonesia) served as the reference iron chelator. Reagents for oxidative stress assays, including thiobarbituric acid (TBA), trichloroacetic acid (TCA), 1,1,3,3‐tetramethoxypropane (MDA standard), glutathione (GSH), glutathione reductase, NADPH, H2O2, potassium ferrocyanide, HCl, and a GPx4 assay kit, were obtained from Sigma‐Aldrich. Tris‐HCl, EDTA, and pyrogallol were supplied by HiMedia Laboratories (Mumbai, India). Histological reagents (buffered formalin, paraffin wax, H&E, and Masson’s trichrome kits) were from BioTessa (Indonesia). Primary antibodies for immunohistochemistry comprised anticollagen type III (Cat. No. PA1‐85323; Invitrogen/Thermo Fisher Scientific, USA), anti‐CD86 (Cat. No. NBP2‐25208; Novus Biologicals, CO, USA), and anti‐CD163 (Cat. No. NB110‐40686; Novus Biologicals), detected with the Elabscience Super Plus IHC Kit (Cat. No. E‐IR‐R221). ELISA kits for IL‐6 (Cat. No. R6000B; R&D Systems, USA), TGF‐β1 (Cat. No. EL‐0162; Elabscience), IL‐10 (Cat. No. E‐EL‐R0016; Elabscience), and MMP‐12 (Cat. No. NBP3‐06764; Novus Biologicals) were used as supplied. All reagents were of analytical or molecular biology grade.

### 2.2. Ethical Approval

All procedures were approved by the Animal Research Ethics Committee of Universitas Padjadjaran, Indonesia (Approval No. 75/UN6.KEP/EC/2024; Registration No. 2210081341) and conducted in accordance with the NRC Guide for the Care and Use of Laboratory Animals and the 3Rs principles. Reporting followed the ARRIVE guidelines.

### 2.3. Experimental Animals

Male Wistar rats (160–180 g; PT Biofarma, Bandung, Indonesia) were acclimated for seven days under controlled conditions (22 ± 2°C, 12‐h light/dark cycle) with standard chow and water provided ad libitum. At study end, animals were anesthetized with ketamine (80 mg/kg i.p.) and euthanized by CO_2_ inhalation.

### 2.4. Preparation of *Caesalpinia Sappan* Extract

*C. sappan* L. wood was obtained from the Forestry and Plantation Service, Yogyakarta, Indonesia; botanical identity was confirmed by a qualified taxonomist (voucher no. KSC001, Herbarium of Padjadjaran University). Air‐dried wood powder was macerated in 96% ethanol for four consecutive 24‐h cycles with intermittent stirring. The filtrate was concentrated under reduced pressure (40°C, rotary evaporator) and dried at 70°C (Memmert UN110) to yield a powder extract, which was passed through an 80‐mesh sieve (180 μm). For administration, SWE was freshly dispersed in distilled water at doses of 25, 50, 75, and 100 mg/kg body weight.

### 2.5. Experimental Design and Tissue Collection

Rats were randomly assigned to seven groups (*n* = 6 each). The normal control group (N; Control) received distilled water throughout. Iron overload was induced in all remaining groups by intraperitoneal injection of ID (15 mg/kg every 3 days for 39 days; cumulative dose of 180 mg/kg BW), a regimen previously validated in our laboratory to produce consistent hepatic oxidative injury and fibrosis. The negative control group (KN; Iron Overload, ID) received ID and distilled water. The positive control group (KP; ID + DFO) received ID and deferasirox (20 mg/kg BW orally, once daily). The four SWE groups (P.1–P.4; ID + SWE 25, 50, 75, and 100) received ID and SWE at 25, 50, 75, or 100 mg/kg BW orally once daily. All treatments were administered for 28 consecutive days. At termination, liver tissues were excised, rinsed in cold saline, and aliquoted for each analytical procedure.

### 2.6. Liver Iron Concentration (LIC)

Hepatic iron concentration (LIC) was determined by atomic absorption spectrophotometry (AAS; AAnalyst 400, PerkinElmer, Germany) at 248.3 nm [18]. Liver tissue (0.2 g) was acid‐digested in concentrated HNO_3_/H_2_O_2_ (30%), diluted to 25 mL, and analyzed in triplicate. Results are expressed as μg Fe/g wet tissue.

### 2.7. Biochemical Assessment of Liver Function

Blood collected by abdominal aortic puncture was centrifuged (3,000 rpm, 15 min) to obtain serum, which was stored at −20°C. Serum ALT, AST, ALP, and albumin were measured photometrically using a Sumifin C1904 semiautomatic analyzer with DiaSys reagents (Holzheim, Germany) according to the manufacturer’s protocol.

### 2.8. Lipid Peroxidation and Antioxidant Enzyme Activities

Hepatic MDA was quantified by the TBARS method [20]. Liver homogenates were treated with 5% TCA, reacted with TBA, and heated at 90°C–100°C for 15–20 min; absorbance was read at 532 nm. Results are expressed as nmol/g wet tissue. SOD activity was determined by inhibition of superoxide radical–mediated reactions and expressed as U/mg protein [21]. GPx4 activity was measured using a Sigma‐Aldrich assay kit; NADPH oxidation was monitored at 340 nm using phosphatidylcholine hydroperoxide as the substrate, and results are expressed as Unit/mg protein, where 1 Unit is defined as the amount of enzyme that oxidizes 1 nmol NADPH per minute [22].

### 2.9. Histopathological Examination

Liver sections (5 μm, paraffin‐embedded) were stained with H&E (hepatocellular morphology), Prussian blue (iron deposition), and Masson’s trichrome (collagen/fibrosis). Slides were examined under an Olympus CX‐23 microscope with a digital camera. Five randomly selected fields per sample were captured and analyzed semiquantitatively with ImageJ (NIH, USA) by an investigator blinded to group allocation.

### 2.10. Immunohistochemistry

Paraffin sections (4 μm) were deparaffinized, rehydrated, and subjected to heat‐induced antigen retrieval. After endogenous peroxidase blocking, sections were incubated overnight at 4°C with primary antibodies against CD86 (M1 marker), CD163 (M2 marker), and collagen type III. Detection was performed using the Elabscience Super Plus IHC Kit with DAB chromogen and hematoxylin counterstaining. Immunoreactive areas were quantified in ImageJ (μm^2^) using a microscope calibration slide.

### 2.11. Quantification of Inflammatory and Fibrogenic Mediators

Hepatic IL‐6, TGF‐β1, IL‐10, and MMP‐12 concentrations were quantified by ELISA using commercially available kits (Section 2.1) according to the manufacturers’ instructions. Liver homogenates were centrifuged (3,000 rpm, 15 min, 4°C); the supernatants were stored at −80°C until further analysis. Absorbance was read at 450 nm, and concentrations were interpolated from standard curves. Results are expressed as pg/mL.

### 2.12. HPLC–PDA Analysis of Brazilin Content

Brazilin in SWE was quantified by HPLC–PDA (Arc system, Waters, USA) on an Eclipse Plus C18 column (250 × 4.6 mm, 5 μm; Agilent, USA) at 40°C. The mobile phase consisted of Solvent A (0.2% formic acid in water) and Solvent B (acetonitrile) under gradient elution (0–9 min, 15% B; 9–12 min, 100% B; 12–16 min, 80% B) at 1.0 mL/min; the injection volume was 10 μL; detection was performed at 286 nm. A brazilin reference standard yielded a linear calibration curve (*r* ^2^ = 0.9998); LOD = 0.2986 μg/mL; LOQ = 0.9048 μg/mL. Results are expressed as mg/g dry extract and % w/w.

### 2.13. Statistical Analysis

Data are presented as mean ± SD. Normality was assessed by the Shapiro–Wilk test; all datasets were normally distributed and were, therefore, analyzed by one‐way ANOVA followed by Duncan’s post hoc test. *p* < 0.05 was considered statistically significant. Analyses were performed using SPSS Version 25 (IBM Corp., Armonk, NY, USA).

## 3. Results

### 3.1. SWE Reduces Hepatic Iron Accumulation

**FIGURE 1:**
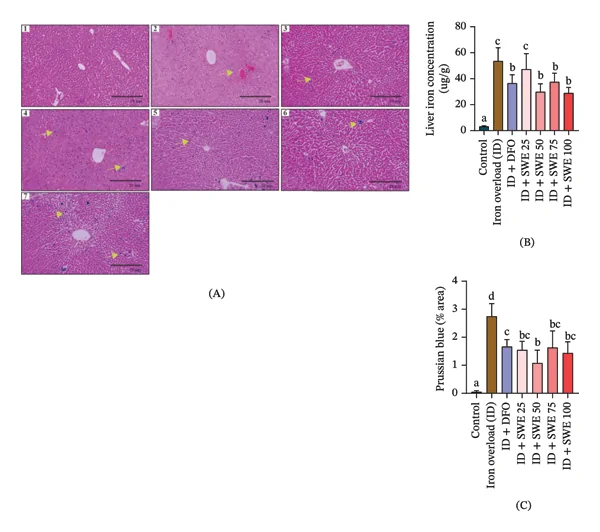
Effects of SWE on hepatic iron accumulation in iron dextran–induced rats. (A) Representative Prussian blue–stained liver sections (400× magnification, scale bar = 20 μm) from Groups 1–7: control, iron overload (ID), ID + DFO, ID + SWE 25, ID + SWE 50, ID + SWE 75, and ID + SWE 100); yellow arrowheads indicate iron‐positive deposits. (B) Quantification of liver iron concentration (LIC) by atomic absorption spectrophotometry (μg Fe/g wet tissue). (C) Semiquantitative analysis of Prussian blue–positive area (% area) by ImageJ. Data are expressed as mean ± SD (*n* = 6). Different letters above bars indicate statistically significant differences between groups (*p* < 0.05, one‐way ANOVA with Duncan’s post hoc test).

ID administration significantly elevated LIC (53.55 ± 10.31 μg/g) and Prussian blue–positive area (2.74 ± 0.46%) in KN relative to normal animals (3.14 ± 0.56 μg/g and 0.05 ± 0.04%, respectively; *p* < 0.05; Figure 1B–C), with dense periportal iron deposits observed in Kupffer cells and hepatocytes (Figure 1A). SWE at doses of 50–100 mg/kg (P.2–P.4) significantly reduced both LIC and Prussian blue–positive area versus KN (*p* < 0.05), whereas P.1 (25 mg/kg) significantly reduced Prussian blue–positive area but not LIC. P.2 (50 mg/kg) and P.4 (100 mg/kg) achieved the greatest LIC reductions (44.24% and 45.99% vs. KN), with P.2 also demonstrating the most pronounced Prussian blue attenuation (60.69%). P.1 (25 mg/kg) showed significant reduction in Prussian blue–positive area but insufficient LIC reduction (47.27 ± 12.02 μg/g; statistically comparable to KN). Deferasirox (KP; 36.49 ± 6.52 μg/g; 39.37% Prussian blue reduction) produced effects comparable to those of P.3–P.4 on both parameters (Figure 1B–C).

### 3.2. SWE Attenuates Iron Overload–Induced Hepatic Dysfunction

**FIGURE 2:**
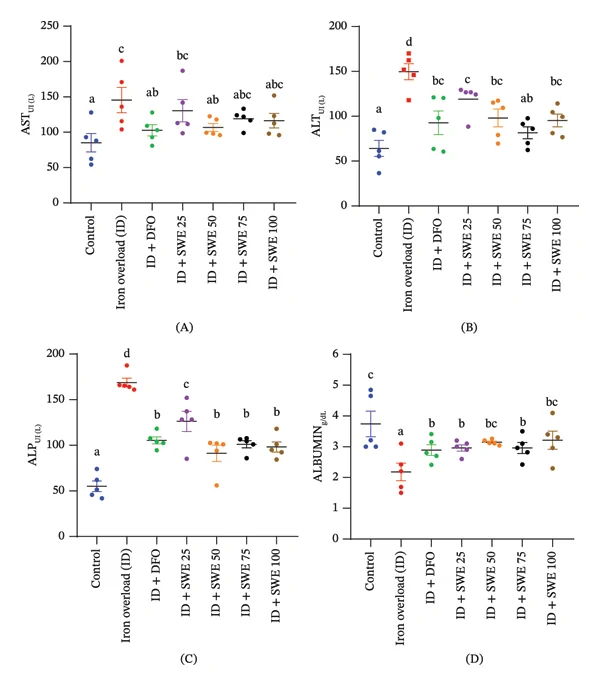
SWE attenuates iron overload–induced hepatic dysfunction. Serum (A) AST, (B) ALT, and (C) ALP activities (IU/L), and (D) albumin concentration (g/dL) across experimental groups. Data are expressed as mean ± SD (*n* = 6 per group). Different letters denote statistically significant differences between groups (*p* < 0.05, one‐way ANOVA, Duncan’s post hoc test).

ID significantly elevated serum AST (145.56 ± 40.09 IU/L), ALT (149.70 ± 20.05 IU/L), and ALP (168.78 ± 10.67 IU/L) in KN versus normal animals (85.15 ± 29.09, 64.09 ± 19.94, and 55.05 ± 13.12 IU/L, respectively; *p* < 0.05; Figure 2A–C), while albumin was significantly reduced (2.18 ± 0.63 vs. 3.74 ± 0.93 g/dL; *p* < 0.05; Figure 2D). SWE significantly reduced all markers versus KN across all doses (*p* < 0.05), with P.2–P.4 producing the greatest transaminase reductions (AST: 26.65%; ALT: up to 45.49% at P.3; ALP: up to 45.98% at P.2) and P.1 producing the least. All SWE groups partially restored albumin (2.96–3.21 g/dL; significantly higher than KN but lower than the normal group; *p* < 0.05). Deferasirox produced comparable effects across all parameters (Figure 2A–D).

### 3.3. SWE Restores GPx4 Activity and Attenuates Lipid Peroxidation

**FIGURE 3:**
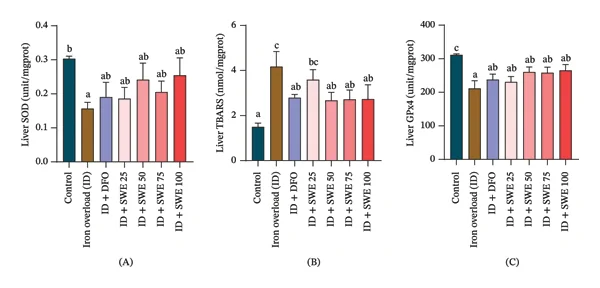
SWE restores GPx4 activity and attenuates lipid peroxidation. Hepatic (A) SOD activity (unit/mg protein), (B) MDA concentration (nmol/mg protein), and (C) GPx4 activity (unit/mg protein) across experimental groups. Data are presented as mean ± SD (*n* = 6 per group). Different letters denote statistically significant differences between groups (*p* < 0.05, one‐way ANOVA, Duncan’s post hoc test).

ID significantly elevated MDA (4.17 ± 1.49 nmol/mg protein) and suppressed SOD (0.16 ± 0.04 unit/mg protein) and GPx4 activity (211.60 ± 51.14 unit/mg protein) in KN versus normal animals (1.50 ± 0.37, 0.30 ± 0.02, and 311.53 ± 6.78, respectively; *p* < 0.05; Figure 3A–C). SWE significantly restored all parameters versus KN across all doses (*p* < 0.05). P.2–P.3 produced the greatest MDA reductions (35.95% and 34.92%), while P.4 yielded the highest GPx4 activity (265.17 ± 39.64 unit/mg protein; 41.82% increase) and SOD recovery (62.83% increase vs. KN). P.1 was least effective (MDA: 13.93% reduction; GPx4: 25.24% increase). Deferasirox produced comparable effects across all three parameters (Figure 3A–C).

### 3.4. SWE Modulates Macrophage Polarization in Iron‐Overloaded Liver

**FIGURE 4:**
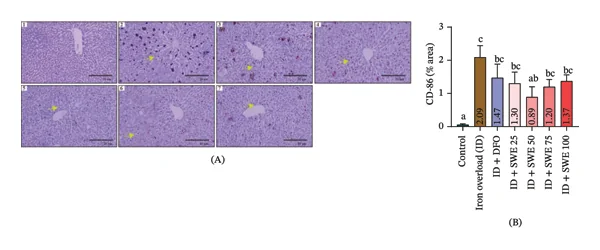
SWE modulates hepatic M1 macrophage polarization in iron‐overloaded liver. Representative CD86 immunohistochemical sections (400× magnification, scale bar = 20 μm) and quantification of CD86 immunoreactive area (% area) across experimental groups. Groups: 1, control; 2, iron overload (ID); 3, ID + DFO; 4, ID + SWE 25; 5, ID + SWE 50; 6, ID + SWE 75; 7, ID + SWE 100. Yellow arrowheads indicate CD86‐immunopositive cells. Data are presented as mean ± SD (*n* = 6 per group). Different letters denote statistically significant differences between groups (*p* < 0.05, one‐way ANOVA, Duncan’s post hoc test).

**FIGURE 5:**
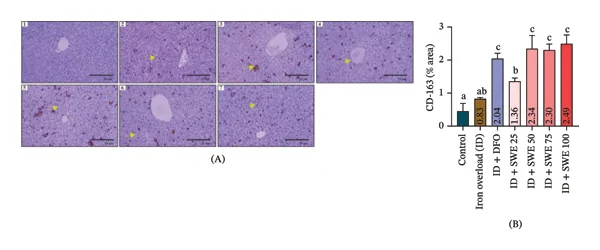
SWE upregulates hepatic M2 macrophage polarization in iron‐overloaded liver. Representative CD163 immunohistochemical sections (400× magnification, scale bar = 20 μm) and quantification of CD163 immunoreactive area (% area) across experimental groups. Groups: 1, control; 2, iron overload (ID); 3, ID + DFO; 4, ID + SWE 25; 5, ID + SWE 50; 6, ID + SWE 75; 7, ID + SWE 100. Yellow arrowheads indicate CD163‐immunopositive cells. Data are presented as mean ± SD (*n* = 6 per group). Different letters denote statistically significant differences between groups (*p* < 0.05, one‐way ANOVA, Duncan’s post hoc test).

ID significantly elevated hepatic CD86 immunoreactivity in KN (2.09 ± 0.79% area) versus normal animals (0.06 ± 0.05% area; *p* < 0.05; Figure 4), indicating predominant M1 activation. CD163 in KN (0.83 ± 0.09%) was slightly elevated versus normal (0.45 ± 0.53%) but did not reach significance (*p* > 0.05; Figure 5). SWE significantly reduced CD86 and upregulated CD163 versus KN at all doses (*p* < 0.05). P.2 achieved the greatest CD86 reduction (57.32% vs. KN), while P.4 produced the highest CD163 upregulation (201.74% vs. KN); P.1 was least effective for both markers (CD86: 37.65%; CD163: 64.22%). Deferasirox produced effects comparable to those of P.2–P.4 on both markers (Figures 4–5).

### 3.5. SWE Regulates Inflammatory and Fibrogenic Mediators

**FIGURE 6:**
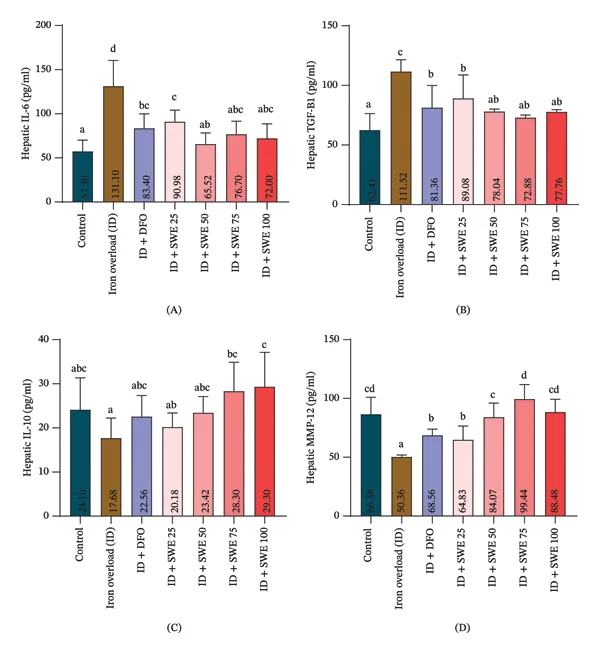
SWE regulates hepatic mediators of inflammation and fibrogenesis. Hepatic concentrations of (A) IL‐6 (pg/mL), (B) TGF‐β1 (pg/mL), (C) IL‐10 (pg/mL), and (D) MMP‐12 (pg/mL) across experimental groups. Data are presented as mean ± SD (*n* = 6 per group). Different letters denote statistically significant differences between groups (*p* < 0.05, one‐way ANOVA, Duncan’s post hoc test).

ID significantly elevated hepatic IL‐6 (131.10 ± 29.36 pg/mL) and TGF‐β1 (111.52 ± 10.00 pg/mL) while reducing IL‐10 (17.68 ± 4.58 pg/mL) and MMP‐12 (50.36 ± 1.56 pg/mL) in KN versus normal animals (57.30 ± 12.72, 62.41 ± 14.06, 24.10 ± 7.26, and 86.38 ± 14.52 pg/mL, respectively; *p* < 0.05; Figure 6A–D). SWE reduced IL‐6 and TGF‐β1 in a dose‐related manner with optimal efficacy at intermediate doses, while increasing IL‐10 and MMP‐12 versus KN across all doses (*p* < 0.05). For IL‐6, P.2 produced the greatest reduction (50.02% vs. KN); for TGF‐β1, P.3 achieved the greatest suppression (34.65%); for IL‐10, P.4 yielded the greatest increase (65.72%); and MMP‐12 recovery was most pronounced with P.3 (97.46%). P.1 was least effective across all four mediators. Deferasirox produced effects comparable to those of most SWE groups but with less pronounced MMP‐12 recovery (36.14% vs. KN; Figure 6A–D).

### 3.6. SWE Attenuates Hepatic Fibrosis

**FIGURE 7:**
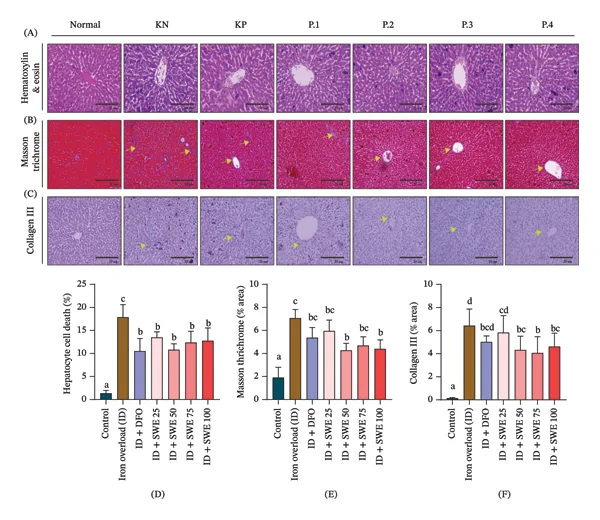
SWE attenuates hepatic fibrosis. Representative liver sections stained with (A) hematoxylin and eosin (H&E), (B) Masson’s trichrome, and (C) collagen III immunohistochemistry across experimental groups (400× magnification, scale bar = 20 μm) (control, iron overload (ID), ID + DFO, ID + SWE 25, ID + SWE 50, ID + SWE 75, ID + SWE 100). Yellow arrowheads indicate areas of collagen deposition and immunopositive cells. Quantification of (D) hepatocyte cell death (% necrotic area by H&E), (E) Masson’s trichrome–positive area (% area), and (F) collagen III immunoreactive area (% area). Data are presented as mean ± SD (*n* = 6 per group). Different letters denote statistically significant differences between groups (*p* < 0.05, one‐way ANOVA, Duncan’s post hoc test).

ID significantly elevated hepatocyte cell death (17.88 ± 2.73%), Masson’s trichrome–positive area (7.07 ± 1.68%), and collagen III immunoreactivity (6.43 ± 1.44%) in KN versus normal animals (1.36 ± 0.61%, 1.90 ± 1.99%, and 0.15 ± 0.05%, respectively; *p* < 0.05; Figure 7D–F). SWE significantly reduced all three parameters versus KN across all doses (*p* < 0.05). P.2 produced the greatest reductions in hepatocyte cell death (39.60%) and Masson’s trichrome area (39.57%), while P.3 achieved the greatest collagen III reduction (36.78%). P.1 was least effective across all parameters. Deferasirox produced comparable reductions to P.2–P.4 across all three endpoints (Figure 7D–F).

### 3.7. HPLC–PDA Identification of Brazilin as the Major Bioactive Constituent of SWE

**FIGURE 8:**
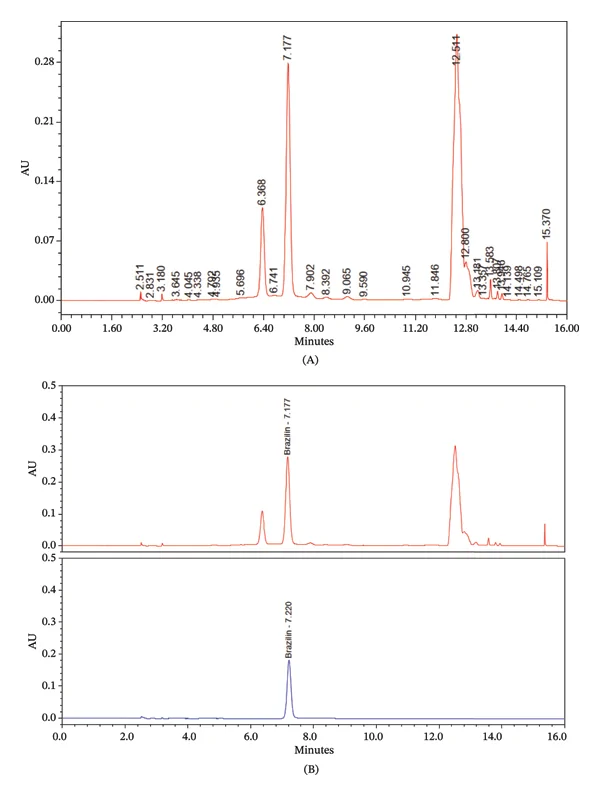
HPLC–PDA identification of brazilin as the major bioactive constituent of SWE. (A) Representative chromatogram of SWE at 286 nm; the dominant peak eluting at 7.177 min corresponds to brazilin. (B) Overlapping chromatograms of SWE (upper, red; retention time 7.177 min) and the brazilin reference standard (lower, blue; retention time 7.220 min), confirming peak identity.

Brazilin was identified as the dominant peak in SWE (retention time 7.177 min), closely corresponding to the reference standard (7.220 min; Figure 8A–B). The calibration curve demonstrated excellent linearity (*r* ^2^ = 0.9998; LOD 0.2986 μg/mL; LOQ 0.9048 μg/mL), yielding a brazilin content of 25.12 ± 0.39% w/w (Figure 8A).

## 4. Discussion

The marked elevation of LIC and Prussian blue–positive area in the KN group, accompanied by significant hepatocellular injury as evidenced by elevated transaminases, reduced albumin, and diffuse periportal iron deposition, confirms the successful induction of hepatic iron overload consistent with prior experimental models employing ID in rodents [23, 24]. Excess hepatic iron promotes the generation of reactive oxygen species via Fenton‐type chemistry, catalyzing lipid peroxidation and disrupting antioxidant defenses, a cascade well‐established in the pathophysiology of iron‐driven hepatocellular injury [9]. In the present study, KN animals exhibited significantly elevated MDA levels alongside markedly suppressed GPx4 and SOD activities, a biochemical signature consistent with ferroptosis‐like oxidative injury. Suppression of GPx4, the canonical phospholipid hydroperoxide‐neutralizing enzyme, renders hepatocytes permissive to uncontrolled lipid peroxidation and membrane rupture, and its reduction under iron overload conditions has been demonstrated both in vitro and in vivo [25]. The concurrent depression of SOD further indicates broad antioxidant collapse rather than pathway‐specific perturbation, consistent with reports of iron overload–induced superoxide dismutase depletion in rodent models [23]. Taken together, the oxidative and functional hepatic parameters in KN animals establish a ferroptosis‐permissive hepatic microenvironment that recapitulates key features of iron‐driven liver fibrogenesis, providing the mechanistic foundation against which the hepatoprotective effects of SWE were evaluated.

SWE treatment exhibited nonmonotonic dose‐response patterns in restoring hepatic GPx4 activity and reducing MDA concentrations across all doses, with the most pronounced antioxidant effects observed at P.2 (50 mg/kg) and P.4 (100 mg/kg), while concurrently improving SOD activity and partially normalizing serum transaminases and albumin. These findings are mechanistically consistent with attenuation of ferroptosis‐like oxidative injury upstream of hepatocyte membrane disruption. The principal bioactive constituent of SWE quantified in the present study brazilin, comprising 25.12 ± 0.39% w/w of the dry extract, has previously been shown to activate Nrf2‐mediated antioxidant signaling through PKC‐dependent Nrf2 nuclear translocation, resulting in upregulation of SOD and glutathione peroxidase activity in an oxidative injury model [26]. The observed biochemical pattern of simultaneous GPx4 recovery, MDA suppression, and SOD restoration is consistent with upstream engagement of the Nrf2–GPx4 axis, as demonstrated in analogous in vivo models of ferroptosis‐associated liver injury [27], although direct mechanistic confirmation through pathway inhibition or target gene expression analysis was not performed in the present study and represents a priority for future investigation. By contrast, deferasirox produced statistically comparable reductions in oxidative stress parameters, an effect most plausibly attributable to its iron‐chelating action that limits substrate availability for Fenton‐driven radical generation rather than to direct antioxidant enzyme induction [23]. The comparable efficacy of SWE and deferasirox across oxidative stress endpoints, achieved through mechanistically distinct routes, suggests that SWE may offer complementary therapeutic value by combining partial iron mobilization with direct antioxidant enzyme induction, a dual mechanism not afforded by iron chelation alone.

The reduction in hepatic CD86 immunoreactivity concurrent with upregulation of CD163 across all SWE doses indicates a functional shift in the hepatic macrophage compartment from proinflammatory M1 toward anti‐inflammatory M2 activation. This interpretation is corroborated by parallel reductions in IL‐6 and TGF‐β1 alongside concurrent increases in IL‐10 and MMP‐12, which together constitute the cytokine signature of M1‐to‐M2 polarization reprogramming in the hepatic microenvironment [14]. Iron overload drives M1 polarization by activating Toll‐like receptor 4 signaling and suppressing the STAT6 pathway, resulting in sustained NF‐κB–mediated CD86 upregulation and IL‐6–driven amplification of hepatic inflammation [12]. Brazilin has been reported to suppress NF‐κB activation and modulate cytokine production in macrophage‐based systems [19], providing a plausible molecular basis for the M1 attenuation observed in the present study. Critically, the present data demonstrate that SWE redirects macrophage polarization without inducing a nonresolving fibrogenic M2 response: TGF‐β1 was significantly suppressed rather than coelevated with IL‐10, thereby distinguishing the observed phenotypic shift from the immunosuppressive M2c subtype characterized by concurrent TGF‐β and IL‐10 elevation [28, 29]. This distinction is clinically meaningful, as an M2c‐dominant hepatic environment would perpetuate stellate cell activation and collagen deposition despite apparent anti‐inflammatory signaling. The highest CD163 and IL‐10 responses were observed at P.3–P.4 (75–100 mg/kg), whereas CD86 suppression and IL‐6 reduction were maximal at P.2 (50 mg/kg), indicating that the anti‐inflammatory and M2‐promoting effects of SWE are not fully dose‐coincident. This dissociation may reflect differential dose‐response thresholds for NF‐κB suppression versus STAT6 activation at the level of macrophage polarization signaling, though mechanistic confirmation requires further investigation [30].

The significant reductions in Masson’s trichrome–positive area and collagen III immunoreactivity in SWE‐treated groups, accompanied by decreased hepatocyte necrosis on H&E staining, are mechanistically supported by concurrent TGF‐β1 suppression and MMP‐12 restoration. TGF‐β1 is the principal fibrogenic cytokine driving hepatic stellate cell transdifferentiation into collagen‐secreting myofibroblasts via the Smad2/3 pathway [31], and its sustained elevation under iron overload represents the central mechanistic link between M1 macrophage activation and progressive extracellular matrix accumulation [25]. SWE‐mediated TGF‐β1 suppression across all doses, most pronounced at P.3 (75 mg/kg; 34.65% reduction vs. KN)—provides a coherent upstream explanation for the reduced collagen III deposition observed histologically. Concurrently, MMP‐12, a macrophage‐derived metalloproteinase with established roles in extracellular matrix degradation and fibrosis resolution [32], was substantially restored by SWE at all doses, most markedly at P.3 (97.46% increase vs. KN), consistent with the M2 polarization shift that preferentially upregulates matrix‐remodeling metalloproteinase expression in alternatively activated macrophages. The convergence of reduced collagen synthesis via TGF‐β1 suppression and enhanced matrix degradation capacity via MMP‐12 restoration constitutes a mechanistically dual antifibrotic profile [33]. This profile distinguishes SWE from deferasirox, which achieved statistically comparable reductions in collagen markers but with substantially less MMP‐12 recovery (36.14% vs. KN), indicating that the antifibrotic mechanism of SWE extends beyond iron chelation to encompass active matrix remodeling, a therapeutically meaningful distinction given that fibrosis resolution, rather than merely its arrest, is the ultimate clinical objective.

Across the full parameter set, the dose‐response pattern of SWE was nonlinear and parameter‐dependent, a finding that warrants mechanistic consideration rather than dismissal as experimental variability. P.2 (50 mg/kg) consistently demonstrated superior performance on oxidative stress markers (GPx4, MDA, SOD), hepatic iron burden (LIC, Prussian blue area), and proinflammatory mediators (IL‐6, CD86), whereas P.3–P.4 (75–100 mg/kg) were more effective at promoting M2 polarization (CD163, IL‐10, MMP‐12) and TGF‐β1 suppression. P.1 (25 mg/kg) was the least effective across all endpoints, indicating that this dose falls below the therapeutically relevant range for this model. The dose‐dissociation between antioxidant and immunomodulatory effects may reflect pathway saturation kinetics: antioxidant‐responsive transcriptional programs such as Nrf2 may reach maximal activation at intermediate doses, while higher brazilin concentrations additionally engage immunomodulatory pathways including STAT6 and PPARγ that preferentially drive M2 polarization [34]. This mechanistic framework, while not yet confirmed in the present study, generates testable hypotheses for subsequent dose‐optimization and target‐engagement investigations. Taken together, the present findings establish SWE standardized to 25.12% w/w brazilin as a multitarget hepatoprotective candidate that simultaneously addresses ferroptosis‐like oxidative injury and macrophage polarization dysregulation, two mechanistically interconnected axes that individually have been targeted in liver fibrosis research but rarely addressed concurrently by a single natural product in a validated in vivo iron overload model.

Several limitations of the present study merit explicit acknowledgment. The exclusive use of male Wistar rats limits generalizability, given established sex‐specific differences in hepatic iron metabolism, macrophage polarization dynamics, and estrogen‐mediated antioxidant signaling. The absence of mechanistic validation tools, including ferroptosis‐specific inhibitors (e.g., ferrostatin‐1 or liproxstatin‐1), flow cytometric characterization of macrophage subpopulations, or analysis of Nrf2 pathway gene expression precludes definitive confirmation of the proposed mechanistic pathways, and the pharmacological observations reported here should be interpreted as pathway‐consistent rather than pathway‐confirmed. Furthermore, while the ID model reproducibly recapitulates transfusional iron overload, it may not fully capture the genetic, metabolic, and inflammatory complexity of human hereditary hemochromatosis or coexisting hepatic pathologies such as metabolic dysfunction‐associated steatotic liver disease. Notwithstanding these limitations, the present study provides the first experimental evidence that SWE simultaneously attenuates ferroptosis‐like oxidative injury and restores macrophage polarization balance in iron overload–induced liver fibrosis, establishing a mechanistic rationale for further preclinical optimization and eventual translational investigation of brazilin‐standardized *C. sappan* preparations in iron‐driven hepatic disease.

## 5. Conclusion

SWE exerts multitarget hepatoprotective effects in iron overload–induced liver fibrosis by simultaneously attenuating GPx4‐centered ferroptosis‐like oxidative injury and restoring macrophage polarization balance from a proinflammatory M1 toward an anti‐inflammatory M2 phenotype. The convergence of suppressed TGF‐β1–driven collagen synthesis and enhanced MMP‐12–mediated matrix degradation mechanistically underlies the observed reductions in hepatic fibrosis and hepatocellular injury. SWE performed comparably to deferasirox across the majority of endpoints while demonstrating superior MMP‐12 restoration, indicating that its hepatoprotective mechanism extends beyond iron chelation to encompass direct antioxidant enzyme induction and immunomodulatory reprogramming. These findings establish the 50–75 mg/kg dose range as the most mechanistically efficacious in this model and provide a rationale for future investigations incorporating Nrf2 pathway confirmation, macrophage subpopulation characterization, and sex‐inclusive experimental designs to advance *C. sappan*–based preparations toward translational evaluation in iron‐driven hepatic disease.

## Author Contributions

Conceptualization: J.N.P. and D.M.M.; methodology: J.N.P. and M.G.; investigation: J.N.P. and E.K.; data curation: J.N.P. and R.S.; formal analysis: J.N.P.; writing original draft: J.N.P.; writing, review and editing: D.M.M. and R.S.; supervision: D.M.M.

## Funding

Padjadjaran University supported this research under the Research Scheme Involving Postgraduate Students.

## Disclosure

All authors have read and approved the final version of the manuscript.

## Conflicts of Interest

The authors declare no conflicts of interest.

## Data Availability

The data that support the findings of this study are available on request from the corresponding author. The data are not publicly available due to privacy or ethical restrictions.
